# PSYCHOSOCIAL WELL-BEING AND COGNITION AMONG OLDER ADULTS IN A LATE LIFE LEARNING PROGRAM IN LEBANON

**DOI:** 10.1093/geroni/igad104.3768

**Published:** 2023-12-21

**Authors:** Peiyi Lu, Martine Elbejjani, Lara Chehabeddine, Hana Arabi, Maya Abi Chahine, Katrina Kezios, Adina Zeki Al Hazzouri, Abla Sibai

**Affiliations:** Columbia University, New York City, New York, United States; American University of Beirut, Beirut, Aakk, Lebanon; American University of Beirut, Beirut, Aakk, Lebanon; American University of Beirut, Beirut, Aakk, Lebanon; American University of Beirut, Beirut, Aakk, Lebanon; Columbia University, New York City, New York, United States; Columbia University, New York City, New York, United States; American University of Beirut, Beirut, Aakk, Lebanon

## Abstract

Growing evidence suggests that engaging in cognitively stimulating activities across the lifecourse is associated with improved health. However, very few studies have evaluated the benefits of late life learning (LLL), especially in the low- and middle-income country setting. We designed a prospective cohort study to evaluate whether LLL is associated with better cognition among older Lebanese and related underlying mechanism. The University for Seniors (UfS) is an established and ongoing LLL program at the American University of Beirut, since 2012, providing cognitively and socially enriching activities (e.g., lectures, study groups, social and cultural events) to older adults, aged 50 and above. Our study will select 700 members (both previous longer-term members and new enrollees) and recruit a comparison group composed of 700 age-, sex-, and education-matched non-UfS-participating community residents. Participants will be interviewed on various topics at baseline and at a two-year follow-up visit. Older Lebanese are exposed to a host of lifecourse psychosocial adversities. We developed a questionnaire to capture their life history such as war-related exposures and economic changes due to recent financial crises. We will also measure participants’ psychological well-being including social network and support, ageism, resilience, hopelessness, loneliness. The challenges of adapting psychosocial scales and capturing lifecourse collective and individual adversities in a particularly burdened low-resourced settings and contextual complexity will be presented. With comprehensive data on LLL and lifecourse adversities, we will be able to examine the potential for scalable low-cost LLL programs to mitigate the impacts of earlier and accumulated psychosocial adversities on cognition.

